# Severe systemic cutaneous adverse reactions following camrelizumab therapy: a case report and literature review

**DOI:** 10.3389/fimmu.2025.1714201

**Published:** 2025-12-19

**Authors:** Yuan-dong Sun, Wen-jia Guo, Jian-jun Han

**Affiliations:** 1 Cancer Research Institute, The Affiliated Cancer Hospital of Xinjiang Medical University, Urumqi, China; 2 Xinjiang Key Laboratory of Translational Biomedical Engineering, Urumqi, China; 3 Center of Interventional Radiology, Shandong Cancer Hospital and Institute Affiliated Shandong First Medical University and Shandong Academy of Medical Sciences, Ji’nan, China

**Keywords:** camrelizumab, immune checkpoint inhibitors, severe cutaneous adverse reaction, squamous cell carcinoma, corticosteroid management

## Abstract

We report a case of a 42-year-old female patient with lip squamous cell carcinoma who developed a severe systemic cutaneous adverse reaction following camrelizumab therapy. The patient had previously undergone wide local excision, chemotherapy, and radiotherapy before receiving camrelizumab in combination with transcatheter arterial chemoembolization. Ten days after the initial infusion, she presented with generalized desquamation, hyperpigmented lesions, and skin breakdown involving the trunk and extremities, most prominently on the hands, arms, and legs. Corticosteroid therapy led to a rapid reduction in pruritus and gradual improvement of cutaneous lesions, with marked healing observed after one week. Remarkably, upon rechallenge with camrelizumab, only mild residual hyperpigmentation was noted without recurrence of severe symptoms. This case highlights the importance of recognizing camrelizumab-associated dermatologic toxicity, emphasizes the role of timely corticosteroid intervention, and suggests that cautious rechallenge may be feasible in selected patients. Further investigation is warranted to elucidate the immunopathological mechanisms underlying severe skin reactions to PD-1 inhibitors and to optimize prevention and management strategies.

## Introduction

Camrelizumab is a humanized monoclonal antibody against programmed death-1 (PD-1) that enhances antitumor immunity and is widely used in the treatment of multiple solid tumors in China (1, 2). Although camrelizumab has demonstrated remarkable antitumor efficacy, its administration may cause severe cutaneous adverse reactions, posing considerable challenges for patients (3). The most common cutaneous adverse reaction to camrelizumab is reactive cutaneous capillary endothelial proliferation (RCCEP), which occurs frequently but is usually mild to moderate; severe skin reactions (≥ grade 3) are overall rare and have only been reported in isolated cases (4). This article presents a case study of a 42-year-old woman with lip cancer who developed a severe systemic cutaneous adverse reaction following camrelizumab treatment, aiming to characterize its clinical features. In addition, we review the literature on skin toxicities associated with PD-1 inhibitors, particularly camrelizumab, to provide context for this adverse event.

## Case report

A 42-year-old woman first presented in January 2024 with a right upper lip mass. Histopathological examination revealed preserved epithelium, multiple clusters of pleomorphic tumor cells within the dermis, keratinized tumor nests, and mild squamous atypia, with infiltration of neutrophils, lymphocytes, and eosinophils, consistent with verrucous carcinoma. She underwent wide excision of the right upper lip lesion with adjacent flap reconstruction. Postoperative pathology demonstrated well-differentiated squamous cell carcinoma (SCC) of the upper lip, invading the dermis and focally abutting the subcutaneous musculature. The excised lesion measured approximately 2.0 × 0.5 cm.

In May 2024, the patient underwent repeat wide excision of a left upper lip lesion with flap transfer and extraction of teeth 22, 23, 34, and 35. Pathology again confirmed SCC. In February 2025, chemotherapy was initiated with cisplatin 20 mg intravenously on day 1, cisplatin 20 mg IV drip on day 2, and cisplatin 10 mg IV drip on day 3. From March 2025, she received radiotherapy to the right upper lip lesion, 5 sessions per week, totaling 33 sessions. The exact radiation dose was not available; however, the patient reported a reduction in dose after eight sessions. Radiotherapy was completed on April 11, 2025. Following completion of radiotherapy, tumor control remained suboptimal, and local skin breakdown developed.

**Figure 1:**
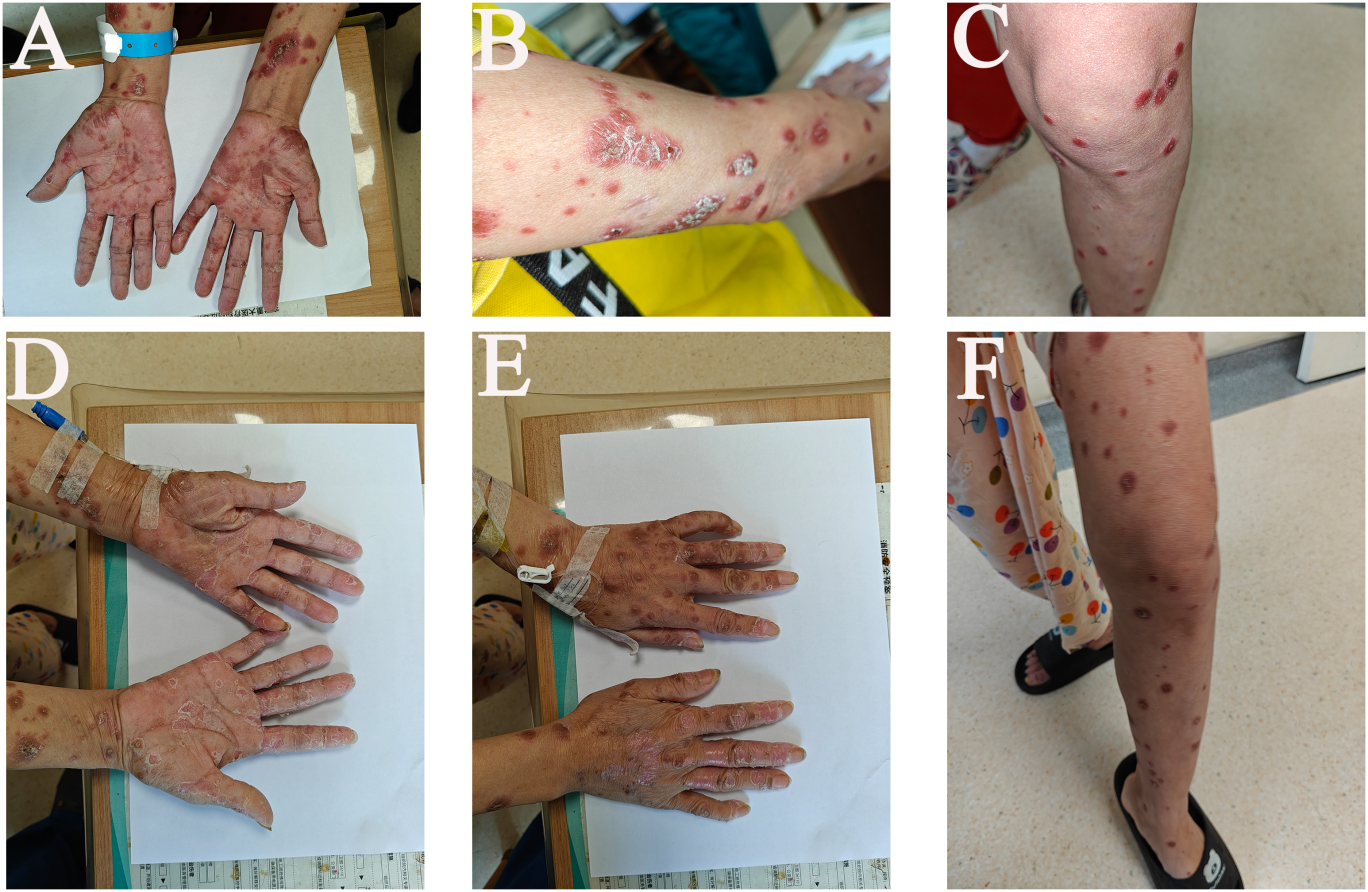
Clinical photographs of severe cutaneous adverse reactions following PD-1 therapy. Ten days after camrelizumab administration: **(A)** hands, **(B)** right upper arm, **(C)** left leg. After three days of corticosteroid treatment: **(D)** hands (palmar surfaces), **(E)** hands (dorsal surfaces), **(F)** left leg.

In June 2025, she underwent transcatheter arterial chemoembolization (TACE) of the right upper lip lesion (showed persistent activity on contrast-enhanced imaging) via the carotid artery, followed by camrelizumab (200 mg, intravenous infusion). On the first day after discharge (the second day after receiving camrelizumab), the patient began to develop skin symptoms, noting mild pruritus and small areas of desquamation on the extremities. By the third day after discharge, the desquamated areas had developed marked hyperpigmentation accompanied by severe pruritus, and both symptoms progressively worsened over the following days. Ten days later, on re-evaluation, she presented with widespread desquamation and hyperpigmented lesions on the trunk and extremities, most severe on the hands, arms, and lower limbs. Some lesions showed skin breakdown with exudation, accompanied by severe pruritus (**Figures 1A–C**). No respiratory, pulmonary, visual, or auditory symptoms were reported.

**Figure 2:**
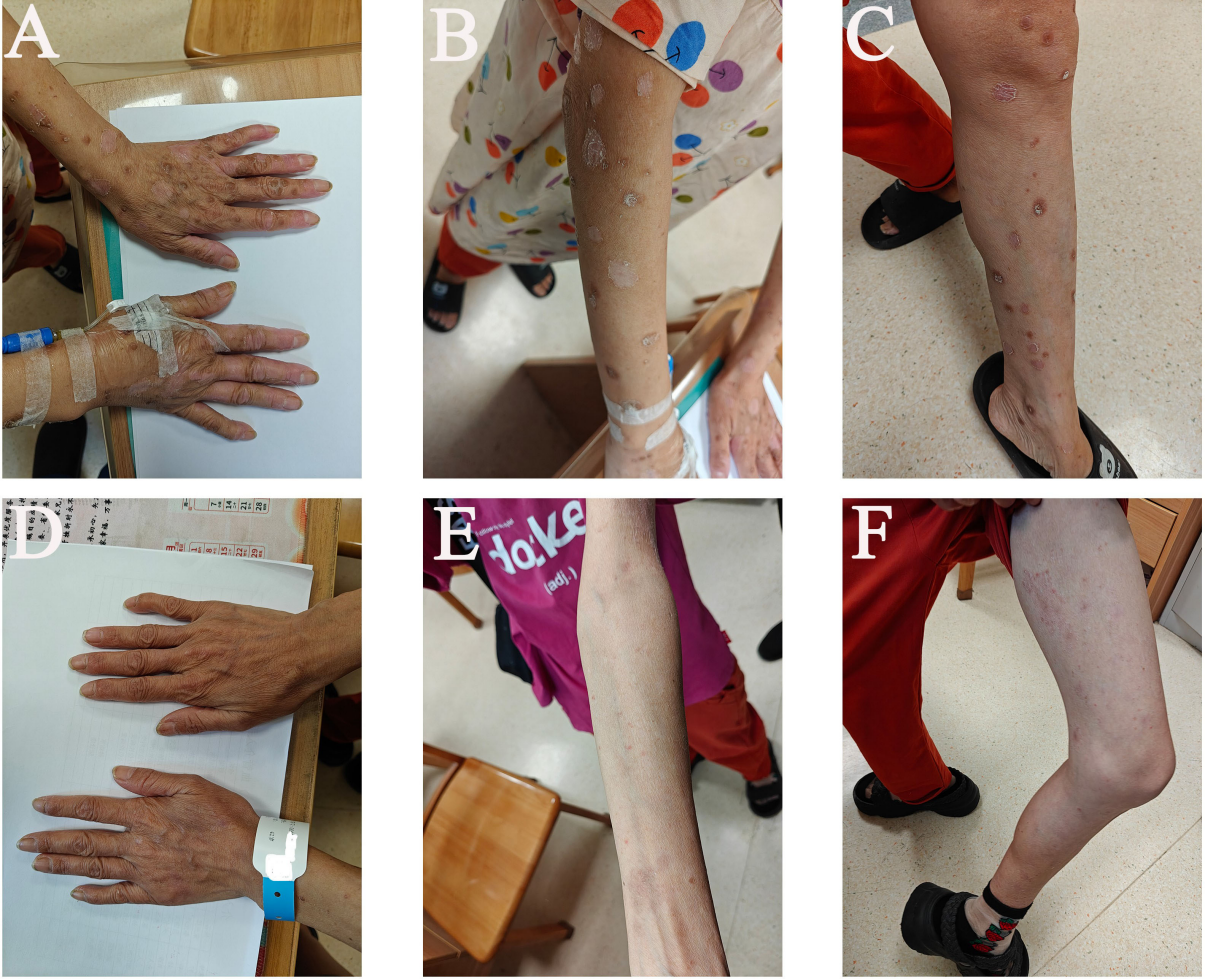
Clinical photographs of severe cutaneous adverse reactions following PD-1 therapy. After seven days of corticosteroid treatment: **(A)** hands, **(B)** right upper arm, **(C)** left leg. Following discontinuation of corticosteroids and re-administration of camrelizumab: **(D)** hands (palmar surfaces), **(E)** left upper arm, **(F)** left leg.

Camrelizumab was withheld, and the patient received intravenous methylprednisolone at 2 mg/kg/day. After four days, pruritus improved significantly, although skin lesions persisted (**Figures 1D–F**). She continued using methylprednisolone for four days after halving the dosage. After eight days of methylprednisolone control, the rash diminished markedly, exudation ceased, and partial healing was observed. At the patient’s request, corticosteroid therapy was discontinued (**Figures 2A–C**). One month after the onset of skin toxicity, the patient requested to resume camrelizumab at the same dose. At that time, only mild residual hyperpigmentation remained without pruritus, desquamation, or skin damage (**Figures 2D–F**).

The patient has since continued maintenance therapy with camrelizumab and remains under regular follow-up, with no recurrence of cutaneous adverse reactions or other discomfort.

**Figure 3:**
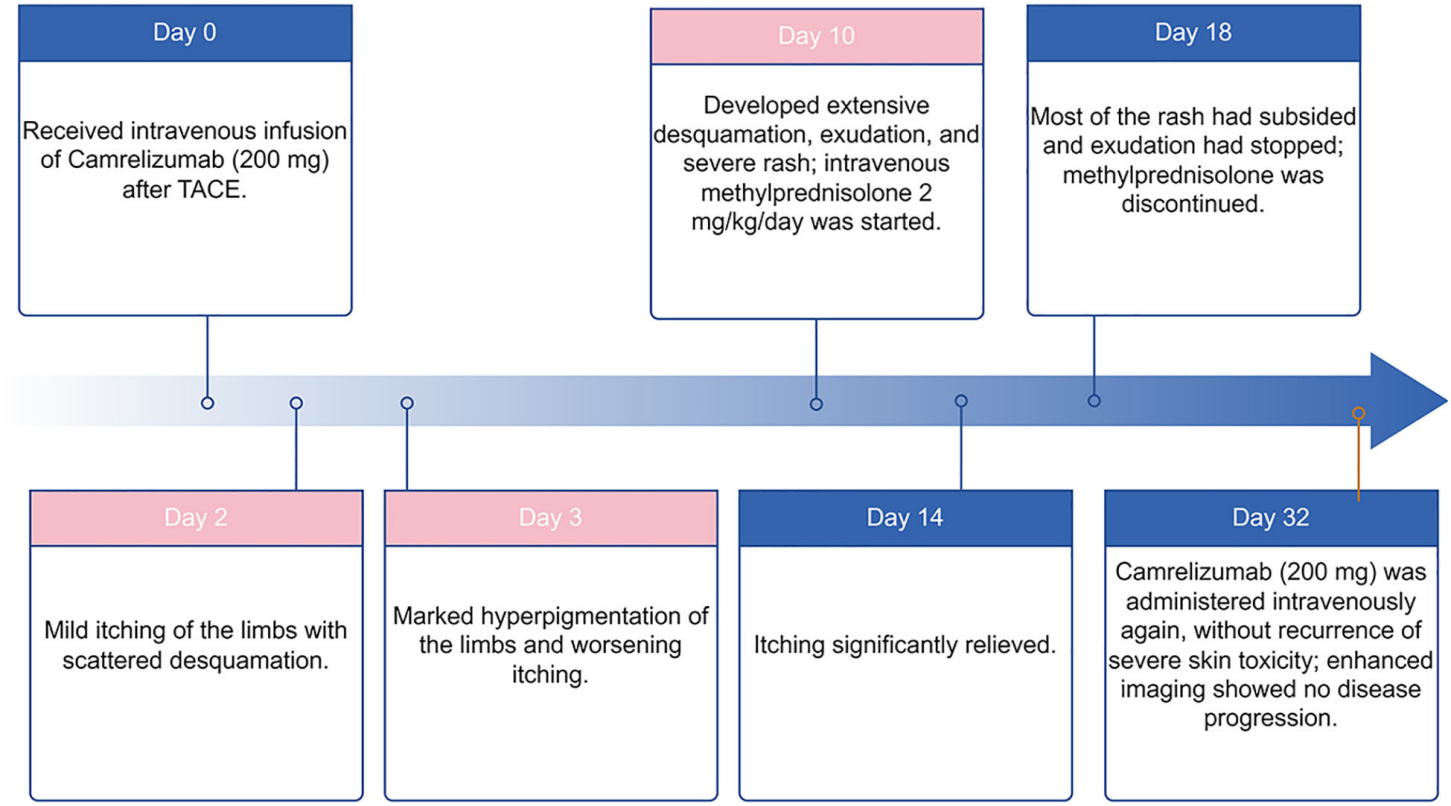
Timeline of camrelizumab administration, cutaneous toxicity onset, management, and outcome. This timeline illustrates the course of camrelizumab treatment, the onset and evolution of cutaneous toxicity, and subsequent management. After receiving camrelizumab (200 mg) on Day 0, the patient developed mild pruritus with scattered desquamation by Day 2, followed by limb hyperpigmentation and worsening itching on Day 3. A severe flare occurred on Day 10, featuring extensive desquamation, exudation, and diffuse rash, leading to initiation of intravenous methylprednisolone (2 mg/kg/day). Symptoms improved markedly by Day 14, and corticosteroids were discontinued by Day 18 once rash and exudation resolved. Camrelizumab was re-administered on Day 32 without recurrence of severe skin toxicity, and imaging confirmed absence of disease progression. Blue panels denote treatment-related events; pink panels indicate cutaneous adverse reactions.

At the time of writing, the patient continues camrelizumab therapy with stable disease control and no further dermatologic toxicity was reported. The patient underwent a repeat contrast-enhanced imaging evaluation of the right upper-lip lesion. The scan demonstrated no further tumor enlargement, no new lesions, and no evidence of local progression compared with the previous examination. The author created a timeline diagram to illustrate the treatment sequence and the occurrence and progression of skin adverse events, in order to provide a clearer overview of the clinical process (**Figure 3**).

## Discussion

The discovery and clinical application of immune checkpoint inhibitors (ICIs) or targeted therapies, particularly PD-1 inhibitors, have profoundly transformed oncology by providing durable therapeutic benefit to many patients. However, with increasing clinical applied, immune-related adverse events have emerged as frequent and clinically significant challenges. The skin and mucosa are the most commonly affected organs, with cutaneous toxicities observed in more than half of patients receiving ICIs, ranging from mild rashes to rare but life-threatening syndromes (5, 6). Most events are low grade and controllable, but their timely recognition and appropriate intervention are critical to safeguard patient safety and maintain treatment efficacy (7–9).

The most common cutaneous adverse reactions include maculopapular rashes and pruritus, typically arising within the first few weeks of treatment (10). ICIs-related cutaneous reactions often show a predilection for the extremities, particularly the hands. These regions contain a dense network of superficial microvasculature and antigen-presenting cells, making them more responsive to systemic immune activation triggered by ICIs. Constant environmental exposure, mechanical friction, and frequent contact with irritants further amplify local inflammation once immune dysregulation begins. As a result, even mild immune activation can manifest more visibly on the hands and lower limbs, leading to earlier onset or more severe presentations in these areas. These manifestations are often self-limiting and respond rapidly to topical corticosteroids and antihistamines, yet they frequently affect appearance and quality of life and may necessitate treatment adjustments. Lichenoid dermatitis, characterized by violaceous papules with mucosal involvement, is strongly associated with PD-1 blockade (11). Psoriasiform and erythrodermic eruptions may represent either new-onset disease or flares of pre-existing psoriasis, sometimes requiring systemic therapy (12). Vitiligo-like depigmentation is particularly evident in melanoma and is generally linked with favorable antitumor responses, highlighting the complex relationship between tumor-directed immunity and host tissue injury (13, 14).

Immune-related cutaneous toxicities from tumor-directed biologics are increasingly understood to arise from dysregulated T-cell activity rather than nonspecific hypersensitivity. PD-1/PD-L1 and CTLA-4 blockade releases inhibitory signals on effector T cells and can reduce regulatory T-cell-mediated tolerance, creating conditions that permit activation of autoreactive clones. In the skin, cytotoxic CD8^+^ T cells and Th1-skewed responses-occasionally with contributions from Th17 pathways—have been implicated in targeting antigens shared between tumor cells and keratinocytes or melanocytes, producing lichenoid interface injury or vitiligo-like depigmentation. Local antigen-presenting cells, interferon signaling, and potential epitope spreading may further amplify tissue inflammation once the reaction is initiated. These immunopathological features help explain why systemic corticosteroids are effective, as they suppress T-cell activation, cytokine production, and tissue infiltration. At the same time, interference with some specific targets related to the skin’s ability to resist damage and recover exacerbates the severity of skin adverse reactions (15). Because many cutaneous irAEs correlate with heightened antitumor immunity in clinical studies, a carefully individualized rechallenge after symptom resolution is often considered feasible, aiming to restore cutaneous tolerance while maintaining therapeutic efficacy.

Bullous autoimmune dermatoses represent another important subset (16). ICI-induced bullous pemphigoid usually begins with intense pruritus and evolves into blistering disease. Though rare, these cases are clinically relevant due to their prolonged course and steroid dependence, with some clinicians advocating methotrexate or dupilumab as steroid-sparing options (17, 18). The most severe cutaneous toxicities are syndromes such as Stevens–Johnson syndrome and toxic epidermal necrolysis, which are rare but life-threatening immune-mediated drug reactions characterized by widespread epidermal necrosis, mucosal involvement, and high risk of fatal complications (19, 20). Although the risk of high-grade cutaneous toxicity is low, management typically requires immediate discontinuation of PD-1 therapy, hospitalization, multidisciplinary consultation, and infection control. Reports suggest that the clinical course of such events under PD-1 inhibition may be more indolent than with classical drugs, but morbidity and mortality remain high.

The mechanisms underlying these toxicities vary but center on alterations in immune tolerance following ICIs therapy. T-cell cross-reactivity against shared antigens in skin and tumor is believed to drive lichenoid and vitiligo-like eruptions, whereas autoantibody production against dermal–epidermal junction proteins underlies bullous pemphigoid (21, 22). Histopathological studies consistently demonstrate cytotoxic T-cell infiltrates, and changes in the cutaneous microbiome and systemic immune environment may further heighten risk. Notably, multiple reports suggest a positive correlation between the occurrence of cutaneous irAEs and improved tumor responses and survival, indicating that these toxicities may represent visible markers of systemic immune reactivation (23–25).

Among PD-1 inhibitors, camrelizumab is uniquely associated with RCCEP, a vascular proliferation observed in over 60% of patients in monotherapy cohorts, most cases being grade 1 or 2 (26). The median onset occurs weeks to months after therapy initiation, and most lesions regress spontaneously. Importantly, retrospective analyses reveal a positive correlation between RCCEP occurrence and superior treatment response and survival, making it both a management challenge and a potential efficacy biomarker (**Table1**) (27, 28).

**Table 1:** Cutaneous adverse events of ICIs: incidence, severity, and management.

Adverse event	Incidence	Severity	Management
Maculopapular rash/Pruritus	20–40% of PD-1/PD-L1 monotherapy patients (33)	Mostly grade 1–2 (9); <5% grade ≥3	Continue ICIs if mild; topical corticosteroids + antihistamines; stop ICIs and oral steroids if ≥grade 2 (34)
Lichenoid dermatitis	5–10% (35)	Grade 1–2, occasionally grade 3	Topical corticosteroids; short courses of oral steroids; dermatology referral if refractory
Psoriasiform/Erythrodermic eruption	~2–5% (new-onset or flare of psoriasis) (36)	Variable; may be severe	Topical corticosteroids, vitamin D analogs; systemic agents (methotrexate, biologics) if refractory
Vitiligo-like depigmentation (esp. melanoma)	5–25% (23, 35)	Cosmetic; not life-threatening	No treatment required; may correlate with better prognosis
Bullous pemphigoid (BP)	<1–2% (36)	Often chronic; may be grade 3–4	Topical/systemic corticosteroids; steroid-sparing agents (methotrexate, dupilumab) (37)
Severe Cutaneous Adverse Reactions (SJS/TEN, DRESS)	<1% (36)	Life-threatening (grade 4–5) (38)	Immediate stop ICIs; hospitalization; systemic steroids ± IVIG/cyclosporine; multidisciplinary care (39)
RCCEP (Camrelizumab)	60–77% (monotherapy); ~24% with apatinib (26)	Mostly grade 1–2; rare grade ≥3 (40)	Observation; local care; usually self-limited; reduced incidence with anti-angiogenic agents

ICIs, Immune Checkpoint Inhibitors; PD-1, Programmed Cell Death Protein-1; PD-L1, Programmed Cell Death Ligand-1; BP, Bullous Pemphigoid; SJS, Stevens–Johnson Syndrome; TEN, Toxic Epidermal Necrolysis; DRESS, Drug Reaction with Eosinophilia and Systemic Symptoms; RCCEP, Reactive Cutaneous Capillary Endothelial Proliferation; IVIG, Intravenous Immunoglobulin.

Management strategies are determined by severity. Mild rashes typically allow continuation of therapy with supportive care. Persistent or widespread grade 2 events often require temporary interruption and oral corticosteroids, whereas severe grade 3 or higher reactions demand systemic immunosuppression, hospitalization, and, in some cases, permanent discontinuation of PD-1 inhibitors. Chronic dermatoses such as bullous pemphigoid may require long-term immunomodulation, and severe reactions may necessitate high-dose systemic corticosteroids, intravenous immunoglobulin, or cyclosporine (29, 30). Across all severities, close collaboration between oncology and dermatology is essential.

Patient education is equally critical, as early recognition and prompt reporting of symptoms substantially reduce morbidity. Rechallenge after resolution of moderate events is feasible in carefully selected patients, with recurrence occurring in approximately one-third but generally at equal or lower severity. Growing evidence underscores the need for improved strategies in prediction and management. Current efforts are focused on identifying predictive biomarkers, including lesional transcriptomic signatures, circulating autoantibodies, and microbiome features. The development of steroid-sparing approaches using targeted biologics is another promising avenue, potentially enabling sustained cancer immunotherapy while reducing chronic toxicity. Long-term follow-up remains essential, as late-onset and persistent cutaneous reactions can occur months after initiation or even after discontinuation of therapy.

The absence of reliable biomarkers makes it difficult to distinguish beneficial immune activation from harmful autoimmunity during treatment. Current management relies heavily on systemic corticosteroids, which may blunt antitumor immunity and lack robust long-term safety data, underscoring the need for steroid-sparing strategies (31, 32). From a translational perspective, deeper integration of dermatology, immunology, oncology, artificial intelligence, and big data research will be critical for refining risk stratification, enabling early intervention, and designing rational therapeutic algorithms. These innovations will allow clinicians to maximize the antitumor efficacy of ICIs while minimizing the risks of cutaneous toxicity.

## Conclusion

Severe cutaneous immune-related adverse events associated with ICIs, while relatively uncommon, demand prompt recognition and carefully tailored management. This case illustrates that early initiation of systemic corticosteroids can effectively control high-grade dermatologic toxicity, and that a judiciously selected rechallenge with camrelizumab may be safely resumed without recurrence. Clinicians should remain alert to atypical or widespread skin manifestations and intervene early to minimize treatment disruption. Further research is warranted to elucidate the underlying immunopathological mechanisms and to refine strategies for the prevention and management of severe cutaneous irAEs.

## Data Availability

The original contributions presented in the study are included in the article/**Supplementary Material**. Further inquiries can be directed to the corresponding authors.
